# FSTVAL: a new web tool to validate bulk flanking sequence tags

**DOI:** 10.1186/1746-4811-8-19

**Published:** 2012-06-18

**Authors:** Joung Sug Kim, Jiye Kim, Tae-Ho Lee, Kyong Mi Jun, Tea Hoon Kim, Yul-Ho Kim, Hyang-Mi Park, Jong-Seong Jeon, Gynheung An, Ung-Han Yoon, Baek Hie Nahm, Yeon-Ki Kim

**Affiliations:** 1 Division of Bioscience and Bioinformatics, Myongji University, Yongin, Kyonggido, 449-728, South Korea; 2 Genomics Genetics Institute, GreenGene BioTech Inc. Yongin, Yongin, Kyonggido, 449-728, South Korea; 3 Upland Crop Research division, National Institute of Crop Science, Suwon, 441-857, South Korea; 4 Graduate School of Biotechnology, Kyung Hee University, Yongin, 446-701, South Korea; 5 National Academy of Agricultural Science, Rural Development Administration, Suwon, 441-707, South Korea

**Keywords:** Flanking Sequence Tags (FSTs), Insertional mutagenesis, Validation, BLAST

## Abstract

**Background:**

Information about a transgene locus is one of the major concerns in transgenic research because expression of the transgene or a gene interrupted by the integration event could be affected. Thus, the flanking sequences obtained from transgenic plants need to be analyzed in terms of genomic context, such as genic and intergenic regions. This process may consist of several steps: 1) elimination of a vector sequence from the flanking sequence, 2) finding the locations in the target genome, and 3) statistics of the integration sites. These steps could be automated for flanking sequences from several dozens of transgenic plants generated in an ordinary targeted gene expression strategy. It would be indispensable in a genome-wide mutagenesis screen using T-DNA or transposons because these projects often generate several thousands of transgenic lines and just as many loci of the transgene among the transgenic plants.

**Results:**

We present an open access web tool, flanking sequence tags validator (FSTVAL), to manage bulk flanking sequence tags (FSTs). FSTVAL automatically evaluates the FSTs and finds the best mapping positions of the FST against a known genome sequence. The statistics, in terms of genic and intergenic regions, are presented as a table, a distribution map, and a frequency graph along the chromosomes. Currently, 17 plant genome sequences, including *Arabidopsis thaliana, Oryza sativa*, and *Glycine max*, are available as reference genomes. We evaluated the utility and accuracy of the tool with 5,144 rice FSTs. The whole process, from uploading the sequences to generating tables of insertions, required a few minutes, with less than 4 clicks in the web environment.

**Conclusions:**

Run for 1 year and tested over 1,000 times, we have confirmed FSTVAL efficiently handles bulk FSTs. FSTVAL is freely available without login at http://bioinfo.mju.ac.kr/fstval/.

## Background

Gene functions can be elucidated by transforming organism by vectors designed for gain or loss of the function. However, although the vectors are carefully designed, the vector components, such as the promoter and terminator, are subject to various gene expression mechanisms involving genetic and epigenetic cell regulation [1], [2]. Moreover, the genomic location of the vector could affect the expression of the transgene. The level or timing of the expression could be varied, whether it is located in euchromatic and heterochromatic regions [3] or it is inserted in promoter, exonic, or intronic regions [4]. For example, the plants whose genomes harbor the transgene within intergenic regions could give unbiased results, as expected from the vector design. Thus, prior to drawing any conclusions from transgenic research, it is routine to test several dozen transgenic plants, as the location of a transgene is very important for understanding the expression of the gene.

Recently, several reverse genetic approaches have been developed to find the function of a gene, such as anti-sense, RNAi suppression, and insertional mutagenesis. These technologies infer a gene function from the phenotype changes of a plant due to blocked or reduced expression of the gene. Among them, insertional mutagenesis by T-DNA or transposon has been generally used for high-throughput functional genomics because of several advantages: random insertion with low copy number in chromosomes, stable inheritance through multiple generations, and high throughput analysis [5]. In Arabidopsis, a dicotyledonous model plant, over 150,000 T-DNA-transformed lines of *Arabidopsis thaliana* were selected, and the precise locations for more than 88,000 T-DNA insertions were determined [6]. In rice, a model plant of monocotyledon, the *Agrobacterium*-mediated co-cultivation method, which is efficient enough to produce a large number of T-DNA insertional mutants, was generally used. An’s laboratory has generated approximately 48,000 T-DNA insertion lines [7,8], while Zhang and colleagues have obtained 31,443 independent T-DNA transformants [9]. As for retrotransposon, approximately 50,000 [10] and 25,000 [11] disruption lines were independently produced using the endogenous retrotransposon Tos17. Rice and Arabidopsis FST-based insertion mutant databases are freely available for searching a mutant of interest (GABI-Kat [12], Salk Institute [6]). For other plant species, such as Chinese cabbage [13], cotton [14], poplar [15], and maize [16,17], similar projects have been developed by large-scale insertional mutagenesis.

To find transgene integration loci, the flanking sequences are obtained by several technologies such as tail-PCR or adaptor mediated PCR. The expression of transgenes in gain-of-function or reverse genetic approaches needs to be analyzed in terms of genomic context (i.e., genic and intergenic regions). If the transgene is inserted in a genic region, the locus has to be further analyzed as to whether it is in a promoter, exon, intron, or terminator. These processes could be automated for flanking sequences from several dozens of transgenic plants generated in an ordinary targeted gene expression strategy. It would be even more important in a genome-wide mutagenesis using T-DNA or transposons because these projects often generate several thousands of transgenic lines.

Several programs have been developed to map cDNA sequences to the genome, such as WebGMAP [18], GeneSeqer [19], and Spaln [20]. Inspired by these programs, we developed flanking sequence tags validator (FSTVAL), an open access tool, to manage bulk Flanking Sequence Tag (FST) mapping via the Internet. FSTVAL automatically evaluates the FSTs and finds the best mapping positions of the FSTs against a known genome sequence. If FSTs include vector sequences, FSTVAL automatically detects those sequences. In addition, FSTVAL provides a distribution map and a frequency graph of FSTs. The entire genome sequence provides the framework for flanking sequence analysis. Currently, 17 organisms, including *Arabidopsis thaliana, Oryza sativa*, and *Glycine max,* are available as reference sequences (Additional file 1). In addition, a user can upload annotated BACs or scaffolds information with sequences to use as a reference in mapping analysis. We tested the utility of FSTVAL using 1,114 and 4,030 FSTs obtained from T-DNA or *Tos17* inserted lines of rice, respectively.

### Implementation

#### FSTVAL software

FSTVAL was built with Django [21], a framework for Web application development, in Python programming language [22], on a mod_wgsi Apache HTTP Server module. Additionally, FSTVAL was developed with basic Web languages such as HTML, CSS, JavaScript, jQuery, and JSON [23] in addition to Python.

Before starting the main analysis, FSTVAL requires FST pre-processing. If uploaded in PHD or FASTQ file formats, the FST file is converted to FASTA format with Bio. SeqIO module in BioPython. During this conversion, low quality sequences are trimmed based on 0.03 minimum error probabilities. This process takes only a few seconds to minutes to complete, depending on the number of FSTs. Next, the FSTs are analyzed by two major modules: the validating module and the mapping module.

In the validating module, the positions of optional sequences, such as border, adaptor, and vector, within FSTs are determined by sequence similarities and qualities. Specifically, the sequence similarities are determined by BLASTN with cutoff values of 10.0, 10.0 and 1e-10 for T-DNA, adaptor, and vector sequence, respectively. Then, FSTs are divided into four types: A (acceptable), NA (not acceptable), Vector, and Low (low quality) based on the similarities and the positions of the optional sequences. Acceptable FSTs are used for the next analysis step.

In the mapping module, FSTs are matched to a genomic sequence by BLASTN or TBLASTX and categorized into six insertional types: exon, intron, 5’upstream, 3’downstream, intergenic, or repeat. The highest scoring region for each FST in the genome sequence is selected as the FST integration site. An FST with the same highest score in several regions of genomic sequence is defined as ‘repeat’. A region between 1,000 bp upstream from the ATG codon and 300 bp downstream from the STOP codon is defined as a genic region [11]. Furthermore, the mapping module produces a graphical distribution map and a frequency graph of FST insertions by PIL (Python Image Library) [24] and Matplotlib [25]. To calculate the frequency, the number of insertions is counted in each 500-kb interval by scanning with a 100-kb sliding window throughout the genomic sequence. The source codes of this software are provided via e-mail by requests.

#### Database architecture

A FSTVAL-specific database was used for categorizing FST insertion sites. The database was constructed as a hierarchical structure with information regarding chromosome, gene, and exon locations and descriptions for 17 plant organisms. For example, one of the rice databases, RAP3 from IRGSP build 3 (Additional file 1), contains 12 chromosomes, 42,057 genes, and 1,611,253 exons that were integrated. Genome annotation in GFF format was parsed and imported into the database. Additional file 1 describes the annotation data used for constructing the database. MYSQL was used to construct the database, which has four tables in its schema. The DATABASE table contains information about species names, annotation versions, annotation associations and file locations of the genome sequence for each species. The CHROMOSOME table contains chromosome numbers and lengths. The GENE table contains gene structure annotations, including 5’-UTR, CDS, and 3’UTR. The EXON table contains start and end positions of each CDS. Since reference database in FSTVAL are easily expanded, the new complete genome sequence and annotated information will be added upon request.

Besides the 17 plants, a user can upload personal BACs or scaffolds sequences file in FASTA format with BED format annotation file as references in mapping analysis. Currently, up to 20 BAC or scaffold sequences of which total length is 200 Mb can be uploaded. To manage the information in the uploaded BED file, SQLite [26] was used as the database management system (DBMS).

## Results

### Data entry and processing

**Figure 1:**
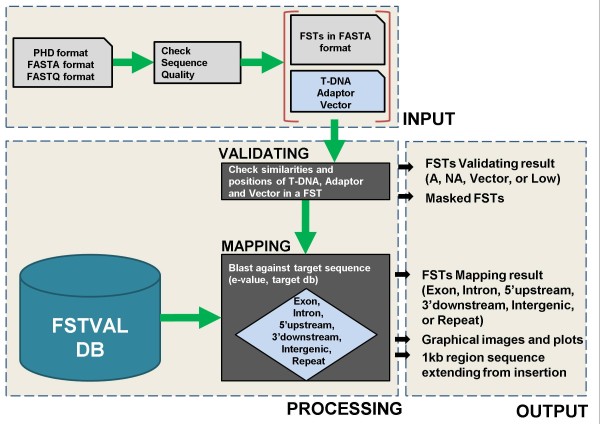
**Procedure in FSTVAL.** FSTVAL involves two consecutive steps: 1.) validate FSTs and 2.) find the best mapping position to the genome. Users can input FSTs in FASTA, FASTQ, or PHD format. First, the regions of the border of T-DNA insertion, the adaptor for PCR amplification, and the binary vector are masked from FSTs, and the FSTs are validated. Next, FSTs are matched to the genome and categorized. The highest scoring region is selected as an FST integration site.

FSTVAL was designed as a web-based tool for analyzing bulk FSTs simultaneously. The tool involves two consecutive steps: validating bulk FSTs and mapping FSTs to genomic DNA. Figure 1 shows all process in the FSTVAL.

First, users paste or upload FSTs in FASTA or FASTQ format or upload a ZIP file containing PHD format files. Optionally, custom data, such as border sequences (i.e., left or right border regions from T-DNA), adaptor sequences from adaptor-ligation PCR methods, and binary vector sequences, can be uploaded. Users can also choose ‘minimum sequence length (MSL)’ excluding the length of border and adaptor sequences (default length is 30 bp).

Next FSTs are divided into four types: A (acceptable), NA (not acceptable), Vector, and Low (low quality) by the FSTVAL validation module. An FST shorter than MSL is indicated as ‘NA’, while an FST larger than MSL is indicated as ‘A’. In the web page, FSTVAL provides the validated result as a downloadable table. In this table, the end position of the border sequence and the start position of the adaptor sequence are presented, as well as the inserted sequences length. In addition, masked sequences can be downloaded, which are denoted as capital and lower-case according to the genomic sequence and the T-DNA border or adaptor sequence, respectively.

Then, acceptable FSTs are matched to the genome by BLASTN or TBLASTX with user-selectable genome database and e-value (or default is 1e-5) [12], and categorized based on their mapping position into six types (exon, intron, 5’upstream, 3’downstream, intergenic, or repeat) by the mapping module. Furthermore, if the insertions occurred in a genic region, the interrupted gene id (which is linked to the GBrowser at its web site) and its description are provided. Additionally, if the FST is mapped to an intergenic region, the information of the nearest gene is provided, given that intergenic regions have been shown to play a role in the expression of adjacent genes and there is increasing evidence that they contain important control sequences [27]. The analysis results can be downloaded as a table.

Lastly FSTVAL provides the distribution map and the frequency of the FST insertions to identify the general distribution of insertions at a glance. The insertion frequency by each chromosome is shown as a table and graph. On the web page, FSTVAL also provides 0.5 kb to 1 kb regions extending 5’ and 3’ from the insertion position (that is denoted by five asterisks). FSTVAL provides a job ID about a result on the top right corner of the web page. If a user remembers the job ID, he/she can find the validating and mapping results. The results will be kept in the server for a month.

### Case study 1: Analysis of 5,144 rice FSTs with FSTVAL

We evaluated the utility of the tool with 1,114 FSTs from T-DNA inserted lines (Additional file 2) and 4,030 FSTs from Tos17 inserted lines (Table 1, Additional file 3) that were generated in japonica rice cultivar ILMI. The flanking regions were isolated by the adaptor-ligation PCR method [9] and then sequenced. For the validation and comparison between T-DNA- and Tos17-flanking sequences, we used PHD and FASTA format, respectively.

**Table 1:** Summary characteristics of T-DNA flanking sequences

**Type of sequences**	**T-DNA FSTs**		***Tos17*FSTs**	
	**No of sequences**	**%**	**No of sequences**	**%**
Acceptable sequence (A) ^a^	706	63.4%	3,921	97.3%
Not acceptable sequence (NA) ^b^	121	10.9%	89	2.2%
vector ^c^	257	23.1%	13	0.3%
Low quality sequence (Low)	30	2.7%	7	0.2%
Total	1,117	100.0%	4,030	100.0%

^a^ FSTs larger than 30 bp after removal of the RB and adaptor sequences.

^b^ FSTs shorter than 30 bp after removal of the RB and adaptor sequences.

^c^ Binary vector or T-DNA tandem repeats.

A total of 1,114 T-DNA FSTs were validated with the border, vector, and the adaptor sequences using a minimum sequence length of 30 bp [12]. Among 1,114 PCR products, 30 (2.7%) had a low quality of sequences, and 963 (86.5%) were passed through the FSTVAL program as readable sequences (Table 1, Additional file 4). A total of 257 sequences (23.1%) corresponded to either T-DNA or binary backbone sequences. Border sequences of T-DNA are associated with head-to-head tandem repeats during *Agrobacterium*-mediated T-DNA transfer [28]. In addition, 'backbone' sequences of a binary vector, as well as T-DNA sequences, are integrated into the genome of plants [29].

Tos17 is a rice endogenous retrotransposon which is activated by tissue culture and is also commonly used for the generation of rice mutants [11]. The amplification of flanking regions from the 3’UTR of Tos17 inserts yielded 4,030 PCR products. Among them, 3,934 (97.6%) were passed as acceptable sequence, except for 89 FSTs shorter than 30 bp and 13 FSTs of original Tos17 position (Table 1, Additional file 5). As PHD formatted files pass through further quality processing steps, the PHD format file had more low-quality sequences than FASTA formatted files.

**Figure 2:**
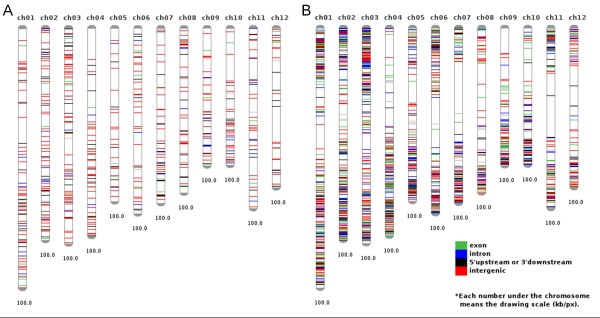
**Distribution maps of the T-DNA (A) and Tos17 (B) FSTs in the rice genome.** Among 1,114 and 4,030 sequences from T-DNA and Tos17 insertion batches, 653 and 3,895 sequences passed through the filtration and chromosome mapping steps, respectively.

**Figure 3:**
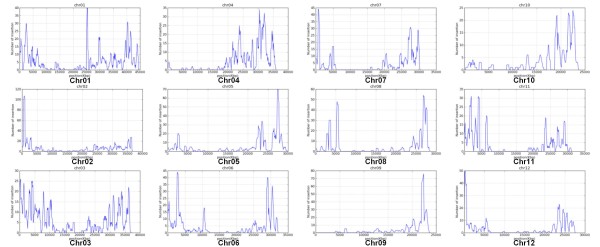
**Frequency graphs of Tos17 insertion on rice chromosomes.** The 3,895 sequences from Tos17 insertion batches are presented as frequencies along the chromosomes.

A total of 706 T-DNA FSTs (63.4%) larger than 30 bp were aligned to the rice genome obtained from RAP 3 (IRGSP build 5) and among them 653 FSTs were positioned in the rice chromosomes. The frequencies of T-DNA insertions in the intergenic and genic regions were 56.4% and 41.8%, respectively (Table 2, Additional file 6). The T-DNA integration frequency is slightly higher in intergenic regions than in genic regions. The frequencies of Tos17 insertions in the genic and intergenic regions were 60.3% and 36.6%, respectively (Table 2, Additional file 7). Tos17 insertion sites were found more often in genic regions and preferably in exon regions (26.4%), compared to the T-DNA insertion sites, which supported previous findings [11]. The distribution of the T-DNA insertion over the 12 rice chromosomes is shown in Table 3. The insertion sites and frequency along the chromosomes are graphically presented in Figures 2 and 3, respectively. Overall, three chromosomes (chr1, chr2, and chr3) were observed to have a high density of T-DNA insertions. As previously shown, chromosomes 5 and 10 had relatively low densities of T-DNA and Tos17 insertions, respectively [7,11]. Tos17 was preferably inserted into the distal end of chromosomes (Figure 2, Figure 3).

**Table 2:** Frequencies of T-DNA insertions in the genic and intergenic regions compared with Tos17

**Type of sequences**		**T-DNA**		***Tos17***	
		**No of sequence**	**Ratio (%)**	**No of sequence**	**Ratio (%)**
Genic		273	41.8%	2,350	60.3%
	Exon	53	8.1%	1,029	26.4%
	intron	56	8.9%	714	18.4%
	5'Upstream(−1,000 bp)	138	21.1%	429	11.0%
	3'Downstream(+300 bp)	26	4.0%	178	4.5%
Intergenic		368	56.4%	1,424	36.6%
Repeat (>1)		12	1.8%	120	3.1%
Total		653	100.0%	3,895	100.0%

**Table 3:** Distribution of T-DNA insertions over the 12 rice chromosomes compared with Tos17 insertions

**Chr. no.**	**Chr. size (Mb)**	**T-DNA**			***Tos17***		
		**No of sequences**	**Ratio (%)**^**a**^	**Average inserts density**^**b**^	**No of sequences**	**Ratio (%)**^**a**^	**Average inserts density**^**b**^
1	43.6	92	14%	0.5	485	13%	11.1
2	35.9	78	12%	0.5	486	12%	13.0
3	36.3	85	13%	0.4	455	12%	12.5
4	35.2	64	10%	0.6	355	9%	10.1
5	29.9	27	4%	1.1	331	9%	11.1
6	31.2	40	6%	0.8	309	8%	9.9
7	29.7	49	8%	0.6	278	7%	9.4
8	28.3	51	8%	0.6	283	7%	10.0
9	23	42	7%	0.5	205	5%	8.9
10	22.9	39	6%	0.6	157	4%	6.9
11	28.5	43	7%	0.7	244	6%	8.6
12	27.5	31	5%	0.9	204	5%	7.4
Total	372.1	641	100%	0.6	3774	100%	10.1

^a^ T-DNA inserts/total inserts.

^b^ Number of T-DNA inserts/size of chromosome.

**Figure 4:**
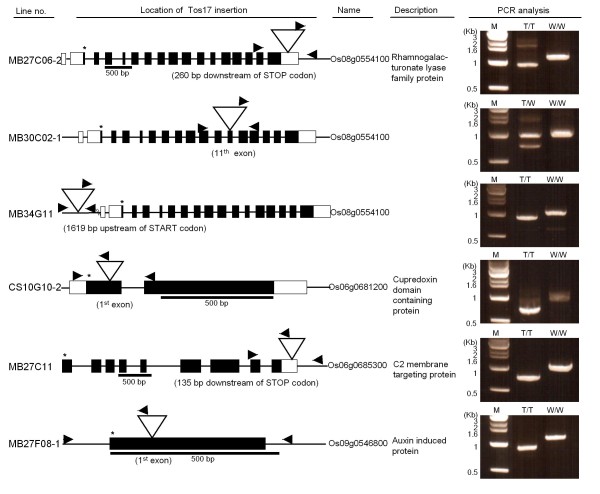
**Identification of Tos17- insertion mutants.** The genomic structures of insertion alleles were determined by FSTVAL analysis in which boxes, bold lines, asterisks, and triangles indicate exons, intron, start codons (ATG), and Tos17, respectively. Scale bar represents DNA length for each gene. The arrow and arrowhead indicate gene specific primer pairs from genomic DNA and 3Tos primers at 239 bp downstream from the 3’ end of Tos17, respectively. Genomic DNA was isolated from the leaf of Tos17 insertion lines for PCR analysis. The PCR product size from wild type was approximately 1 kb, which is 240 bp smaller than that from Tos17 insertion lines. W/W wild type, T/W heterozygous for Tos17 insertion line, T/T homozygous for Tos17 insertion line.

To confirm the accuracy of FSTVAL analysis, six lines were selected from Tos17 insertion lines FSTVAL showed that lines such as MB27C06-2, MB30C02-1, and MB34G11 have Tos17 insertion in 3’UTR, exon, and promoter (1.6 kb upstream of the start codon) regions of Os08g0554100, respectively. In CS10G10-2, MB27F08-1, and MB27C11 lines, Tos17 was inserted in the exon of Os06g0681200 Os09g0546800, and 3’UTR of Os06g0685300, respectively (Figure 4). Tos17 insertion was examined by PCR from genomic DNA. Gene specific primer pairs were designed within a 1-kb region extending 5’ and 3’ from the insertion positions provided by FSTVAL (Additional file 8, Additional file 9). PCR fragments were amplified with gene specific primers and a Tos17 primer designed at 239 bp upstream from the 3’ end of Tos17. The PCR product size from Tos17 insertion lines was smaller than that obtained from wild type (Figure 4, Additional file 9). This result showed that the prediction of the position of Tos17 insertion through FSTVAL is identical to the PCR result.

We also tested the first 200 sequences from T-DNA sequences (Additional file 2) and analyzed them in our FSTVAL and a publicly available program “WebGMAP”. Among 200 sequences, 189 sequences gave the same annotated information suggesting both programs can be used to give genome locus information (data not shown).

In the 5,144 FST analyses, the average FSTVAL processing time was 19 min in the web server having AMD Phenom II X4 Quad-Core processor with 2.5 GHz clock speed.

### Case study 2: Analysis massive FSTs by FSTVAL

If the number of FSTs is higher than 10,000, the graphical presentation might be limited because all of the chromosomes are tainted with lines of insertion marks (Additional file 10). In consideration of this case, we provide a frequency graph as well as a distribution map. We tested 27,621 preexisting rice T-DNA insertion sequences (Additional file 11) that are publically available [30] and presented them as a frequency graph (Additional file 12). FSTVAL showed an equal performance with a large number of insertion sequences as with several dozen sequences.

## Discussion

FSTVAL is designed to map FSTs to the genome via a web-based interface. All of the procedures from loading FSTs to blasting against released public genome data are performed through the web. If tagged sequences contain vector sequences, FSTVAL detects those. Statistics of transgene loci are summarized in a table and frequency figures along the chromosomes. Our analysis using in-house generated 1,114 and 4,030 FSTs from T-DNA or Tos17 inserted lines of rice, respectively, highlights the applicability of FSTVAL. Further analysis using preexisting rice T-DNA insertion sequences (27,621) showed FSTVAL can be applicable even in large-scale insertional mutagenesis screens such as those on-going in Chinese cabbage [13], cotton [14], poplar [15], and maize [16,17].

In-house generated T-DNA and Tos17 lines showed that FSTVAL handled not only the statistics of insertion sites and visualization of these along the chromosomes, but was also helpful in the analysis in lines that have a transgene in distinct regions of a gene. As shown, three lines have Tos17 inserted into the 3’UTR, exon, and promoter regions of Os08g0554100 (a MYST1 protein), respectively. PCR amplification and sequencing of these fragments were consistent with the results as predicted by FSTVAL. In-depth analysis of these lines is preceded by a selection of homo lines from these plants.

Transgene expression in plants is highly variable, even among plants independently transformed with the same construct. Many factors may be responsible for variable transgene expression, including transgene copy number, position effect, DNA methylation, and the repetitiveness of the transgene insert. It is therefore important to produce transgenic plants as many as possible. FSTVAL is expected to be efficiently used in these procedures.

Efforts to obtain information on transgene loci will be important to genome-wide research using T-DNA and retrotransposons. Since the first plant genome, *Arabidopsis thaliana*, was sequenced in the year 2000 [31], numerous plant genomes, such as rice [32-34], poplar [35], *Glycine max*[36], and *Sorghum bicolor*[37], have been sequenced. Moreover, with the rapid improvement of sequencing techniques, more plant genome sequences are near completion and waiting for public release. However, the functions of many genes in these plant genomes are predicted by similarity with expressed genes such as ESTs or by gene finding algorithms such as GLIMMER [38] and GENSCAN [39] trained using the same ESTs. Genome-wide mutagenesis using T-DNA and retrotransposons might be invaluable resources to identify the functions of these genes. These projects will generate many transgenic plants and nearly as many flanking sequences around the insertions. The functionality of FSTVAL might be applicable to genome-wide mutagenesis.

## Conclusions

FSTVAL is designed to conveniently analyze FSTs ranging from a few dozen to several tens of thousands of occurrences. The first web-based tool for validation of massive flanking sequences through any browser, FSTVAL offers an automatic evaluation of flanking sequences, whether they contain sequences other than genomic sequences, and the best mapping position of the FST against a genome. We tested the utility of FSTVAL with FSTs from T-DNA or *Tos17* inserted lines of rice and the robustness was proved by running for a year and testing over 1,000 times. At present, 17 genome sequences, including rice, Arabidopsis, and maize, are currently available as reference sequences, and more plant genome information will be integrated. Additionally, for a genome which is not being served but genome information is available a user can analyze it by uploading the genome sequence and annotated information.

### Availability and requirements

Project name: FSTVAL

Project home page: http://bioinfo.mju.ac.kr/fstval/

Operating system(s): Platform independent

Programming language: Python, JavaScript, HTML, etc.

## Abbreviations

FSTVAL, Flanking Sequence Tags Validator; FST, Flanking Sequence Tag; MSL, Minimum Sequence Length.

## Competing interests

The authors declare that they have no competing interests.

## Authors’ contributions

JSK, JK, THL and KMJ designed the software architecture, and JSK and JK wrote most of this paper. JK, THL, and THK built a database and implemented the software. YHK, HMP, JSJ and GA provided flanking sequences for testing the utility of FSTVAL. BHN and YKK inspired the overall work and revised the final manuscript. All authors read and approved the final manuscript.

## Supplementary Material

Additional file 1 Data used for constructing FSTVAL database. Click here for file

Additional file 2 Masked FSTs from T-DNA sequences. Click here for file

Additional file 3 Masked FSTs from Tos17 sequences. Click here for file

Additional file 4 The validating result of T-DNA flanking sequences. Click here for file

Additional file 5 The validating result of Tos17 flanking sequences. Click here for file

Additional file 6 The mapping result of T-DNA flanking sequences. Click here for file

Additional file 7 The mapping result of Tos17 flanking sequences. Click here for file

Additional file 8 1 kb regions extending 5’ and 3’ from the Tos17 insertion position. Click here for file

Additional file 9 PCR primers. Click here for file

Additional file 10 Distribution maps of massive FSTs. 27,621 preexisting rice T-DNA insertion sequences were mapped on the rice chromosomes. Click here for file

Additional file 11 27,621 T-DNA sequences. Click here for file

Additional file 12 Frequency graphs of massive FSTs. 27,621 preexisting rice T-DNA insertion sequences were presented as frequencies along the chromosomes. Click here for file
