# Supplementary material for: FSTVAL: a new web tool to validate bulk flanking sequence tags
Source: Plant Methods. 2012 Jun 18;8:19. doi: 10.1186/1746-4811-8-19 (PMC3439307; doi:10.1186/1746-4811-8-19)
Supplement: Additional file 1 — Data used for constructing FSTVAL database. [file 1746-4811-8-19-S1.pdf]

Additional file 1. Data used for constructing FSTVAL database.

Scientific name	Common name	Source	Version	Reference
Arabidopsis lyrata	Lyrate rockcress	JGI	JGI release v1.0	[1]
Arabidopsis thaliana	Arabidopsis	TAIR	TAIR10.0	[2]
Brachypodium distachyon	Purple false brome	JGI	JGI v1.0 8x assembly, MIPS/JGI v1.0 annotation	[3]
Carica papaya	Papaya	ASGPB	December 2007	[4]
Chlamydomonas reinhardtii	Green algae	JGI	v4.3 release	[5]
Cucumis sativus	Cucumber	JGI	Phytozome v7.0	[6]
Glycine max	Soybean	JGI	Glyma1 release	[3]
Oryza sativa	Rice	MSU	MSU release 6.1	[7]
	Rice	RAP-DB	IRGSP build 5	[8]
Physcomitrella patens	Moss	JGI	version 1.6	[9]
Populus trichocarpa	Western poplar	JGI	version 2.0	[10]
Ricinus communis	Castor bean plant	JCVI	TIGR/JCVI release v0.1	[11]
Sorghum bicolor	Cereal grass	JGI	v1.0 release (Sbi1 assembly, Sbi1.4 gene set)	[12]
Theobroma cacao	Cacao	USDA -ARS	Release 1.0	[13]
Vitis vinifera	Grape vine	Genoscope	March 2010 release	[14]
Volvox carteri	Volvox	JGI	Phytozome v7.0	[15]
Zea mays	Maize	Maizesequence	Release 5a	[16]
Brassica rapa	Chinese cabbabge	BRAD	Release v1.1	[17]

Reference

1. Hu TT, Pattyn P, Bakker EG, Cao J, Cheng JF, Clark RM, Fahlgren N, Fawcett JA, Grimwood J, Gundlach H *et al*: **The Arabidopsis lyrata genome sequence and the basis of rapid genome size change**. *Nature genetics* 2011, **43**(5):476-481.
2. Swarbreck D, Wilks C, Lamesch P, Berardini TZ, Garcia-Hernandez M, Foerster H, Li D, Meyer T, Muller R, Ploetz L *et al*: **The Arabidopsis Information Resource (TAIR): gene structure and function annotation**. *Nucleic acids research* 2008, **36**(Database issue):D1009-1014.
3. Schmutz J, Cannon SB, Schlueter J, Ma J, Mitros T, Nelson W, Hyten DL, Song Q, Thelen JJ, Cheng J *et al*: **Genome sequence of the palaeopolyploid soybean**. *Nature* 2010, **463**(7278):178-183.
4. Ming R, Hou S, Feng Y, Yu Q, Dionne-Laporte A, Saw JH, Senin P, Wang W, Ly BV, Lewis KL *et al*: **The draft genome of the transgenic tropical fruit tree papaya (Carica papaya Linnaeus)**. *Nature* 2008, **452**(7190):991-996.
5. Merchant SS, Prochnik SE, Vallon O, Harris EH, Karpowicz SJ, Witman GB, Terry A, Salamov A, Fritz-Laylin LK, Marechal-Drouard L *et al*: **The Chlamydomonas genome reveals the evolution of key animal and plant functions**. *Science* 2007, **318**(5848):245-250.
6. Huang S, Li R, Zhang Z, Li L, Gu X, Fan W, Lucas WJ, Wang X, Xie B, Ni P *et al*: **The genome of the cucumber, Cucumis sativus L**. *Nat Genet* 2009, **41**(12):1275-1281.
7. Ouyang S, Zhu W, Hamilton J, Lin H, Campbell M, Childs K, Thibaud-Nissen F, Malek RL, Lee Y, Zheng L *et al*: **The TIGR Rice Genome Annotation Resource: improvements and new features**. *Nucleic acids research* 2007, **35**(Database issue):D883-887.
8. Rice Annotation P, Itoh T, Tanaka T, Barrero RA, Yamasaki C, Fujii Y, Hilton PB, Antonio BA, Aono H, Apweiler R *et al*: **Curated genome annotation of Oryza sativa ssp. japonica and comparative genome analysis with Arabidopsis thaliana**. *Genome research* 2007, **17**(2):175-183.
9. Rensing SA, Lang D, Zimmer AD, Terry A, Salamov A, Shapiro H, Nishiyama T, Perroud PF, Lindquist EA, Kamisugi Y *et al*: **The Physcomitrella genome reveals evolutionary insights into the conquest of land by plants**. *Science* 2008, **319**(5859):64-69.
10. Tuskan GA, Difazio S, Jansson S, Bohlmann J, Grigoriev I, Hellsten U, Putnam N, Ralph S, Rombauts S, Salamov A *et al*: **The genome of black cottonwood, Populus trichocarpa (Torr. & Gray)**. *Science (New York, NY)* 2006, **313**(5793):1596-1604.
11. Chan AP, Crabtree J, Zhao Q, Lorenzi H, Orvis J, Puiu D, Melake-Berhan A, Jones KM, Redman J, Chen G *et al*: **Draft genome sequence of the oilseed species Ricinus communis**. *Nature biotechnology* 2010, **28**(9):951-956.
12. Paterson AH, Bowers JE, Bruggmann R, Dubchak I, Grimwood J, Gundlach H, Haberer G, Hellsten U, Mitros T, Poliakov A *et al*: **The Sorghum bicolor genome and the diversification of grasses**. *Nature* 2009, **457**(7229):551-556.

13. Argout X, Salse J, Aury JM, Guiltinan MJ, Droc G, Gouzy J, Allegre M, Chaparro C, Legavre T, Maximova SN *et al*: **The genome of Theobroma cacao**. *Nature genetics* 2011, **43**(2):101-108.
14. Jaillon O, Aury JM, Noel B, Policriti A, Clepet C, Casagrande A, Choisne N, Aubourg S, Vitulo N, Jubin C *et al*: **The grapevine genome sequence suggests ancestral hexaploidization in major angiosperm phyla**. *Nature* 2007, **449**(7161):463-467.
15. Prochnik SE, Umen J, Nedelcu AM, Hallmann A, Miller SM, Nishii I, Ferris P, Kuo A, Mitros T, Fritz-Laylin LK *et al*: **Genomic analysis of organismal complexity in the multicellular green alga Volvox carteri**. *Science* 2010, **329**(5988):223-226.
16. Schnable PS, Ware D, Fulton RS, Stein JC, Wei F, Pasternak S, Liang C, Zhang J, Fulton L, Graves TA *et al*: **The B73 maize genome: complexity, diversity, and dynamics**. *Science* 2009, **326**(5956):1112-1115.
17. Cheng F, Liu S, Wu J, Fang L, Sun S, Liu B, Li P, Hua W, Wang X: **BRAD, the genetics and genomics database for Brassica plants**. *BMC plant biology* 2011, **11**:136.
