# Supplementary figures and images for: FSTVAL: a new web tool to validate bulk flanking sequence tags
Source: Plant Methods. 2012 Jun 18;8:19. doi: 10.1186/1746-4811-8-19 (PMC3439307; doi:10.1186/1746-4811-8-19)

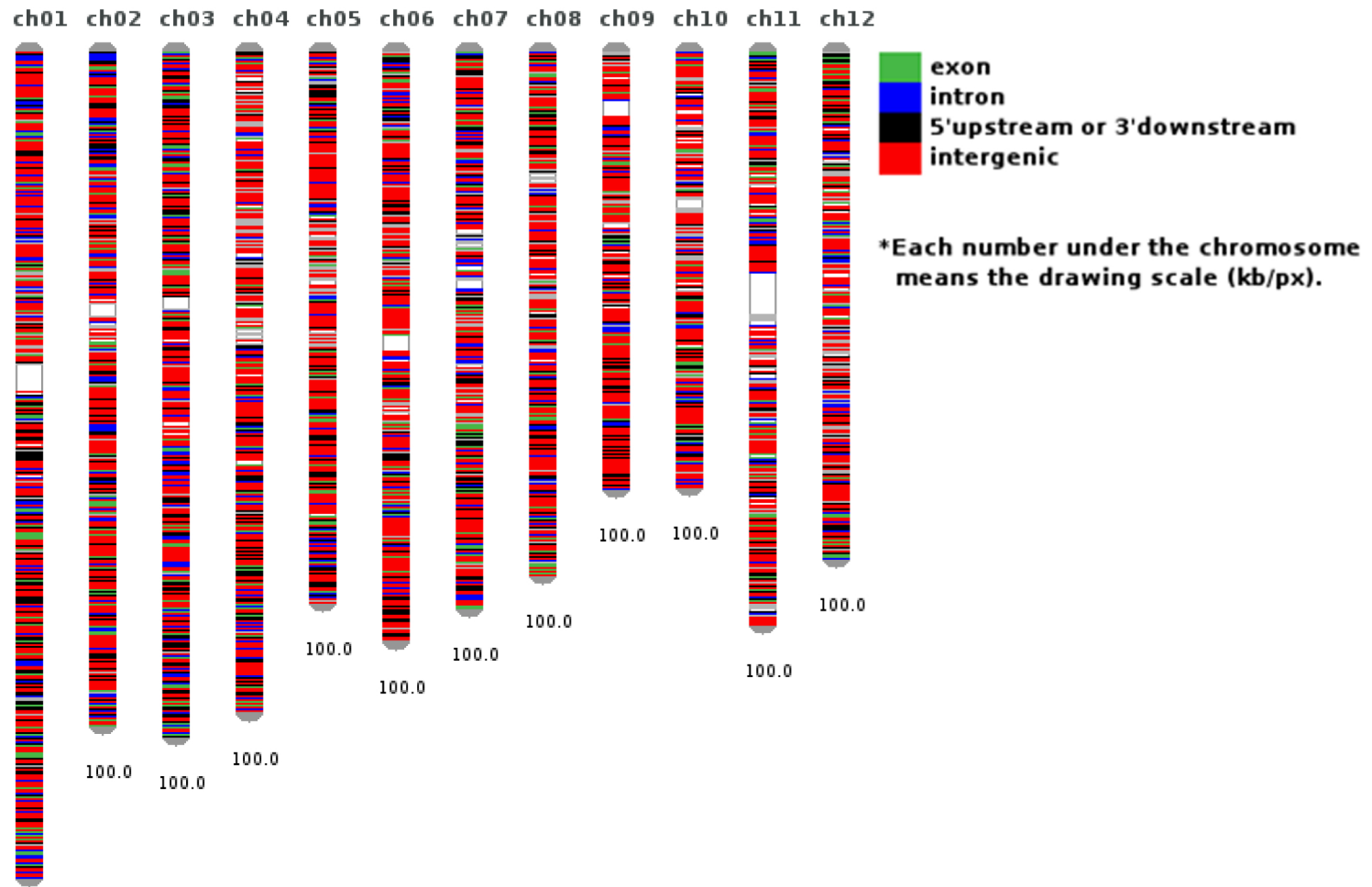

**Additional file 10. Distribution maps of 27,621 T-DNA sequences in the rice chromosome.**

Supplement: Additional file 10 — Distribution maps of massive FSTs. 27,621 preexisting rice T-DNA insertion sequences were mapped on the rice chromosomes. [file 1746-4811-8-19-S10.pdf]

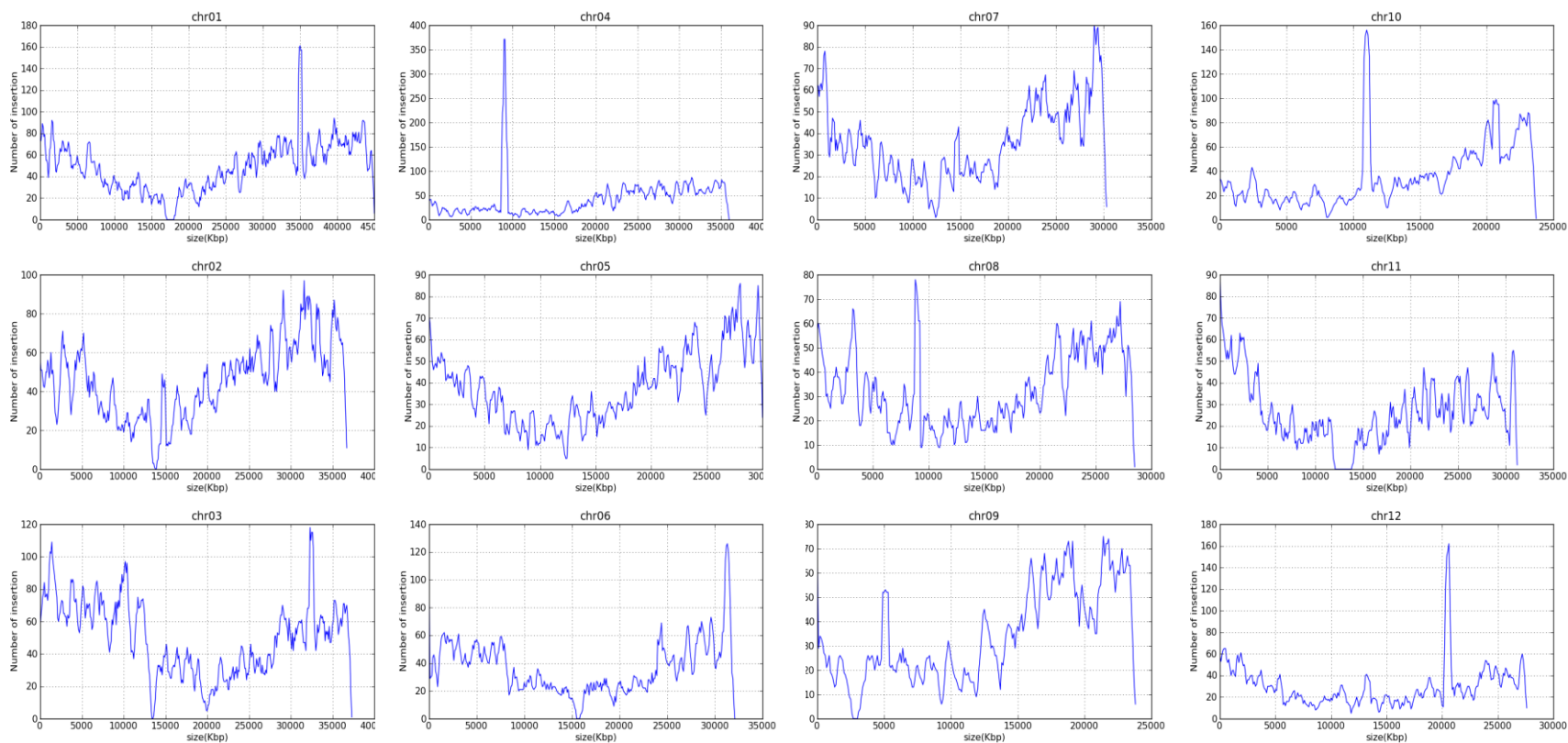

**Supplementary Figure 2: Frequency graphs of rice 27,621 T-DNA sequences on rice chromosome.**

Supplement: Additional file 12 — Frequency graphs of massive FSTs. 27,621 preexisting rice T-DNA insertion sequences were presented as frequencies along the chromosomes. [file 1746-4811-8-19-S12.pdf]
